# Induced proximity of a TIR signaling domain on a plant-mammalian NLR chimera activates defense in plants

**DOI:** 10.1073/pnas.2001185117

**Published:** 2020-07-24

**Authors:** Zane Duxbury, Shanshan Wang, Craig I. MacKenzie, Jeannette L. Tenthorey, Xiaoxiao Zhang, Sung Un Huh, Lanxi Hu, Lionel Hill, Pok Man Ngou, Pingtao Ding, Jian Chen, Yan Ma, Hailong Guo, Baptiste Castel, Panagiotis N. Moschou, Maud Bernoux, Peter N. Dodds, Russell E. Vance, Jonathan D. G. Jones

**Affiliations:** ^a^The Sainsbury Laboratory, University of East Anglia, NR4 7UH Norwich, United Kingdom;; ^b^Department of Molecular and Cell Biology, and Cancer Research Laboratory, University of California, Berkeley, CA 94720;; ^c^Agriculture and Food, Commonwealth Scientific and Industrial Research Organisation, Canberra, ACT 2601, Australia;; ^d^Department of Biology, Kunsan National University, 54150 Gunsan, South Korea;; ^e^John Innes Center, NR4 7UH Norwich, United Kingdom;; ^f^Department of Plant Biology, Uppsala BioCenter, Swedish University of Agricultural Sciences and Linnean Center for Plant Biology, SE-756 61 Uppsala, Sweden;; ^g^Department of Biology, University of Crete, GR 71 500 Heraklion, Crete, Greece;; ^h^Institute of Molecular Biology and Biotechnology, Foundation for Research and Technology - Hellas, GR 70 013 Heraklion, Crete, Greece; ^i^HHMI, University of California, Berkeley, CA 94720

**Keywords:** NLR immune receptors, plant immunity, inflammasome, effector-triggered immunity

## Abstract

Plant and animal intracellular nucleotide-binding, leucine-rich repeat (NLR) immune receptors detect pathogen-derived molecules and activate defense. Plant NLRs can be divided into several classes based upon their N-terminal signaling domains, including TIR (Toll-like, Interleukin-1 receptor, Resistance protein)- and CC (coiled-coil)-NLRs. Upon ligand detection, mammalian NAIP and NLRC4 NLRs oligomerize, forming an inflammasome that induces proximity of its N-terminal signaling domains. Recently, a plant CC-NLR was revealed to form an inflammasome-like hetero-oligomer. To further investigate plant NLR signaling mechanisms, we fused the N-terminal TIR domain of several plant NLRs to the N terminus of NLRC4. Inflammasome-dependent induced proximity of the TIR domain in planta initiated defense signaling. Thus, induced proximity of a plant TIR domain imposed by oligomerization of a mammalian inflammasome is sufficient to activate authentic plant defense. Ligand detection and inflammasome formation is maintained when the known components of the NLRC4 inflammasome is transferred across kingdoms, indicating that NLRC4 complex can robustly function without any additional mammalian proteins. Additionally, we found NADase activity of a plant TIR domain is necessary for plant defense activation, but NADase activity of a mammalian or a bacterial TIR is not sufficient to activate defense in plants.

## Significance

Animal NLRs form wheel-like structures called inflammasomes upon perception of pathogen-associated molecules. The induced proximity of the signaling domains at the center of the wheel is hypothesized to recruit caspases for the first step of immune signal transduction. We expressed a plant-animal NLR fusion to demonstrate that induced proximity of TIR signaling domains from plant NLRs is sufficient to activate plant immune signaling. This demonstrates that a signaling-competent inflammasome can be formed from known, minimal components. The intrinsic NADase activity of plant TIRs is necessary for immune signaling, but fusions to a bacterial or a mammalian TIR domain with NADase activity, which also lead to accumulation of NAD^+^ hydrolysis products (e.g. cyclic ADP-ribose), were unable to activate immune signaling.

Plants lack an adaptive immune system and rely on innate immunity to defend against pathogens (1, 2). Upon perception of pathogen effector proteins by plant nucleotide-binding, leucine-rich repeat (NLR) intracellular receptors, immune signaling is initiated that often culminates in a programmed cell death called the hypersensitive response (HR) (3). NLRs carry an N-terminal signaling domain, a nucleotide-binding (NB) domain, and a C-terminal leucine-rich repeat (LRR) domain (2). The P-loop (or Walker A) motif contained in the NB domain binds ATP or ADP. NLRs typically bind ADP in their resting state and exchange it for ATP when activated (1, 2, 4). The two major classes of plant NLRs are defined by the N-terminal signaling domain they contain: either a TIR (Toll-like, Interleukin-1 receptor, Resistance protein) or a CC (coiled-coil) domain (2). Ectopic expression of TIR and CC domains can activate immune signaling (1, 5–7). Self-association interfaces are required for TIR-mediated immune signaling (8–10), but the mechanism of signaling activation is unknown.

Animal NLR domain architecture resembles plant NLRs (1, 2, 11). NLRC4, a mammalian NLR, contains an N-terminal caspase activation and recruitment domain (CARD), a NACHT NB domain, and a C-terminal LRR. NLRC4 cooperates with NAIP NLRs to detect bacterial PAMPs (pathogen-associated molecular patterns); in mice, NAIP1 and NAIP2 detect type III secretion system (T3SS) needle and rod components, respectively, and NAIP5 detects flagellin (12, 13). The PAMP binds to the NAIP protein, altering its conformation and provoking recruitment of an NLRC4 molecule, which initiates stepwise recruitment of additional NLRC4 molecules, forming a wheel-like oligomer called an inflammasome (14–16). This complex brings the N-terminal CARDs into close proximity, allowing recruitment and activation of caspases (1, 17). Hence, activation of NLRC4-mediated immune signaling occurs via induced proximity of N-terminal signaling domains.

Recently, the structure of *Arabidopsis* ZAR1, an NLR with a CC N-terminal signaling domain, was resolved in complex with the pseudokinase RKS1 and the decoy kinase PBL2 (18, 19). ZAR1 associates with RKS1, and the effector AvrAC uridylylates PBL2 and induces its recruitment to the ZAR1-RKS1 heterodimer (20). Subsequently, a wheel-like structure forms, termed a “resistosome,” consisting of five heterotrimeric ZAR1-RKS1-PBL2 protomers, and activates an immune response (18, 19, 21). Similar to the NLRC4-inflammasome, induced proximity is imposed on the N-terminal CC signaling domains leading to a significant structural change in this domain. This suggests that induced proximity of N-terminal signaling domains may be a conserved mechanism of signaling activation in NLRs, although it has not yet been observed in TIR-domain containing NLRs. Here, we fused the TIR domain from RPS4, a well-characterized *Arabidopsis* NLR (22–24), to NLRC4 to investigate whether induced proximity imposed by an animal NLR is sufficient to activate an N-terminal TIR signaling domain of a plant NLR in planta.

Some but not all TIR domains can hydrolyze NAD^+^ to nicotinamide and various forms of ADP ribose (ADPR) (25–28). A conserved catalytic glutamate is required for NAD^+^ hydrolysis (25, 26). This catalytic glutamate is also required for defense activation for plant TIRs (22, 27). Plant and bacterial TIRs, in contrast to the TIR domain of mammalian SARM1, can make a variant cyclic ADPR (v-cADPR) (25, 27, 28). Here, we use the TIR-NLRC4 platform to demonstrate that while NADase activity of plant TIRs is necessary for their activation of cell death, the in vivo generation of v-cADPR or cADPR is not sufficient to induce cell death.

## Results and Discussion

**Fig. 1.**
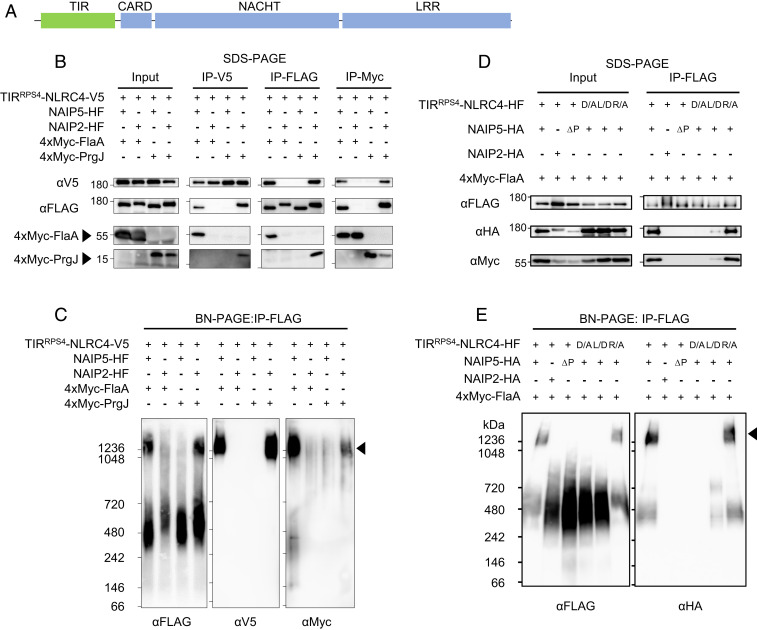
Induced proximity of TIR^RPS4^ triggers HR-like cell death when fused to NLRC4. (*A*) Schematic diagram of domain architecture of TIR^RPS4^-NLRC4 drawn to scale. TIR domain, Toll-like, interleukin-1 receptor, Resistance protein domain; CARD, caspase activation and recruitment domain; NACHT, NAIP, CIITA, HET-E, TP1; LRR, leucine rich repeat. TIR^RPS4^ contains amino acids residues 1–236 of RPS4. (*B* and *C*) Oligomerization assay to test the formation of inflammasome-like complexes in plants. *N. benthamiana* leaves were transiently cotransformed with combinations of TIR^RPS4^-NLRC4, NAIP, PAMP (as indicated by a + or – symbol), and silencing suppressor p19 by *A. tumefaciens* infiltration. After 3 d, leaves were harvested, proteins tagged with a FLAG epitope were immunoprecipitated and subjected to SDS-PAGE (*B*) and BN-PAGE (*C*), and immunoblotted for V5, FLAG or Myc. Results shown are representative of at least three independent replicates. See also *SI Appendix*, Fig. S1. Arrowhead indicates predicted inflammasome complex. HF, (His)_6_-(FLAG)_3_ tag. SDS-PAGE (*D*) and BN-PAGE (*E*) of mutant versions of TIR^RPS4^-NLRC4 or NAIP5. TIR^RPS4^-NLRC4, NAIP, and FlaA were immunoprecipitated from extracts from coinfiltrated *N. benthamiana* leaves. Results shown are representative of at least three independent replicates. D/A and L/D indicate NLRC4 mutations D125A and L435D, respectively; R/A indicates TIR^RPS4^ mutation R116A; ΔP indicates NAIP5 with a deleted P-loop (amino acids 464–487; NAIP5ΔPloop). Arrowhead indicates predicted inflammasome complex.

We examined whether NLRC4-imposed induced proximity is sufficient to activate defense mediated by the RPS4 TIR domain (TIR^RPS4^). We generated a TIR^RPS4^-NLRC4 chimaera under control of the CaMV 35S promoter for expression in plant leaves (Fig. 1*A*). To determine if this construct could form a TIR^RPS4^-NLRC4/NAIP/PAMP inflammasome in plants, we transiently coexpressed epitope-tagged TIR^RPS4^-NLRC4, NAIP2, NAIP5, and either *Legionella pneumophila* flagellin (FlaA) or *Salmonella typhimurium* T3SS rod protein PrgJ in *Nicotiana benthamiana*. To detect inflammasomes, we separated proteins extracted from infiltrated leaves by blue native polyacrylamide gel electrophoresis (BN-PAGE) (*SI Appendix*, Fig. S1). We additionally purified the complex by immunoprecipitating NAIPs to remove background immunoblot signal (Fig. 1 *B* and *C*). Upon coexpression in leaves of TIR^RPS4^-NLRC4, NAIPs, and PAMPs, all inflammasome proteins interacted when in cognate combinations: NAIP5 and FlaA, or NAIP2 and PrgJ, but not in other NAIP-ligand combinations (Fig. 1*B*).

An oligomeric complex of >1,200 kDa, consistent with an inflammasome, also appeared upon coexpression in leaves of TIR^RPS4^-NLRC4 and cognate NAIP-PAMP pairs (Fig. 1*C*).

NLRC4 has two interaction surfaces that mediate oligomerization: the “donor” surface, which recruits NLRC4 monomers to the complex, and the “acceptor” surface, which interacts with the donor surface of other NLRC4s and NAIPs (14–16). We transiently coexpressed either an acceptor surface mutant (D125A) or a donor surface mutant (L435D) of TIR^RPS4^-NLRC4 with NAIP5 and FlaA. As expected, the acceptor surface mutant TIR^RPS4^-NLRC4(D125A) did not immunoprecipitate with NAIP5 (Fig. 1*D*), nor was it capable of forming an inflammasome, because the acceptor surface was prevented from associating with the donor surface of NAIP5 (Fig. 1*E*). The donor surface mutant TIR^RPS4^-NLRC4(L435D) did immunoprecipitate with NAIP5 but did not form a >1,200 kDa inflammasome (Fig. 1*E*). NAIP5 and FlaA immunoprecipitated with TIR^RPS4^-NLRC4(L435D) at a lower apparent affinity than with TIR^RPS4^-NLRC4 (Fig. 1*D*). This may be due to an avidity defect: A fully assembled inflammasome ring likely increases the apparent affinity of all members of the complex. Therefore, the TIR^RPS4^-NLRC4(L435D)/NAIP5/FlaA trimer may dissociate more readily than the full ∼13-mer inflammasome. Consistent with previous results (12), a P-loop motif deletion in NAIP5 prevented in planta inflammasome assembly (Fig. 1 *D* and *E*). Taken together, these results indicate that TIR^RPS4^-NLRC4/NAIP/PAMP can form an authentic inflammasome complex in plants.

**Fig. 2.**
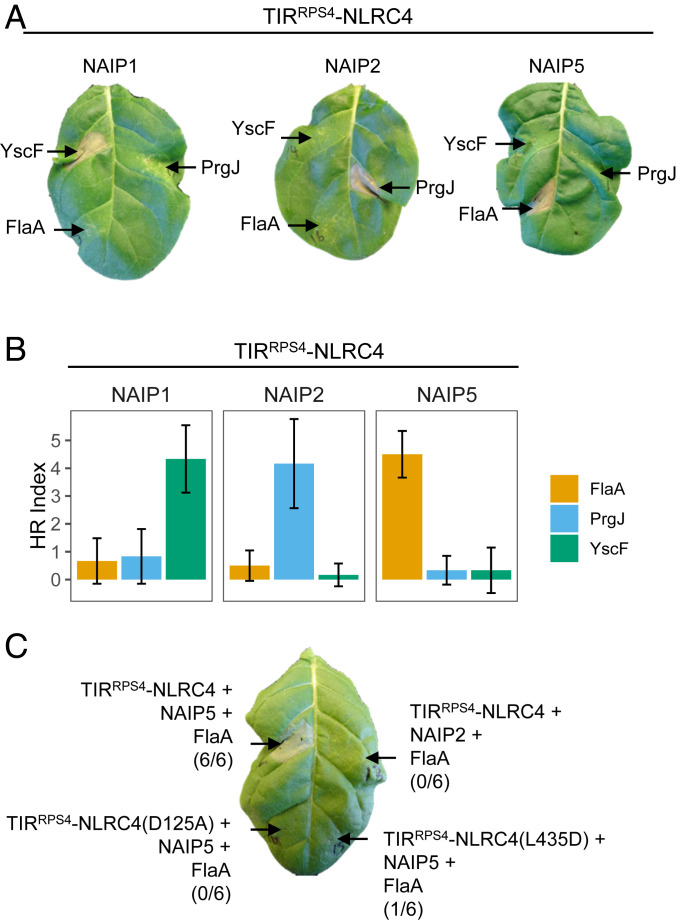
TIR^RPS4^-NLRC4 inflammasome oligomerization triggers HR-like cell death. (*A*) HR-like cell death is elicited by coexpression of RPS4^TIR^-NLRC4 and inflammasome components that oligomerize. *N. tabacum* leaf sections were coinfiltrated with *A. tumefaciens* strains (each at OD_600_ = 0.5) carrying RPS4^TIR^-NLRC4, a NAIP, and a PAMP. HR was visually assessed and scored after 3 days postinfiltration (dpi). (*B*) Mean HR index of visually assessed tobacco infiltrations. Each graph represents coinfiltrations of a different NAIP with TIR^RPS4^-NLRC4 and three ligands (indicated on the *x* axis), with a sample size of *n* = 6. Error bars show SD. HR index scale shown in *SI Appendix*, Fig. S2*F*. (*C*) HR cell death assay of NLRC4 interface mutants. *N. tabacum* leaf sections were coinfiltrated with NAIP5-HA, 4xMyc-FlaA, and TIR^RPS4^-NLRC4-HF, or donor or acceptor surface mutants of TIR^RPS4^-NLRC4-HF. HR was visually assessed and photographed after 3 dpi. The numbers in parentheses are the number of leaves displaying HR equivalent to the image shown out of the total number of leaves infiltrated.

We next asked whether a mammalian inflammasome signaling platform could activate TIR-dependent plant responses, like HR. Transient coexpression of the following inflammasome-forming combination triggered HR in *N. tabacum* leaves: TIR^RPS4^-NLRC4, NAIP1, and the T3SS needle protein from *Yersinia pestis* (YscF); TIR^RPS4^-NLRC4/NAIP2/PrgJ; and TIR^RPS4^-NLRC4/NAIP5/FlaA (Fig. 2 *A* and *B*; HR index scale shown in *SI Appendix*, Fig. S2*F*). No cell death was observed when combinations that do not form inflammasomes were coexpressed (Fig. 2 *A* and *B*). TIR^RPS4^-NLRC4 donor and acceptor surface mutants, which are incapable of forming inflammasomes, coexpressed with NAIP5 and FlaA did not trigger HR (Fig. 2*C*); this indicates that a higher-order complex containing multiple TIR domains is required, and not just a conformational change in TIR^RPS4^-NLRC4 induced by interaction with NAIP5/FlaA. Similarly, NAIP5 P-loop mutants did not trigger HR when expressed with TIR^RPS4^-NLRC4 and FlaA (*SI Appendix*, Fig. S2*A*). These data suggest that induced proximity of the RPS4 TIR domain is sufficient to activate immune signaling and HR.

Structure-function analyses of TIR domains revealed two surfaces of the RPS4 TIR essential for RPS4-mediated immunity: The AE interface surface, required for heterodimerization and homodimerization, and the DE surface, predicted to be a self-interaction surface (8, 9). To test whether inflammasome-mediated signaling acts by promoting self-association through these interfaces, we introduced an AE interface mutation (S33A, H34A; SH/AA) and a DE interface mutation (R116A) into separate TIR^RPS4^-NLRC4 constructs. NAIP5 and FlaA formed inflammasomes with TIR^RPS4(SH/AA)^-NLRC4 and TIR^RPS4(R116A)^-NLRC4 (Fig. 1 *D* and *E* and *SI Appendix*, Fig. S2 *B* and *C*). Inflammasomes containing TIR^RPS4(SH/AA)^-NLRC4 exhibited no HR and TIR^RPS4(R116A)^-NLRC4 exhibited occasional, weak HR (*SI Appendix*, Fig. S2 *D* and *E*). Taken together, these data demonstrate that oligomerization via the NLRC4-NACHT was not sufficient for TIR^RPS4^ HR and that both TIR self-association interfaces are required. Wan et al. and Horsefield et al. reached similar conclusions by fusing TIR^RPS4^ to the SAM oligomerization domain from SARM1 (27, 28). Hence, induced proximity promotes formation of a TIR domain oligomer that is sufficient for signaling through the AE and DE interfaces.

**Fig. 3.**
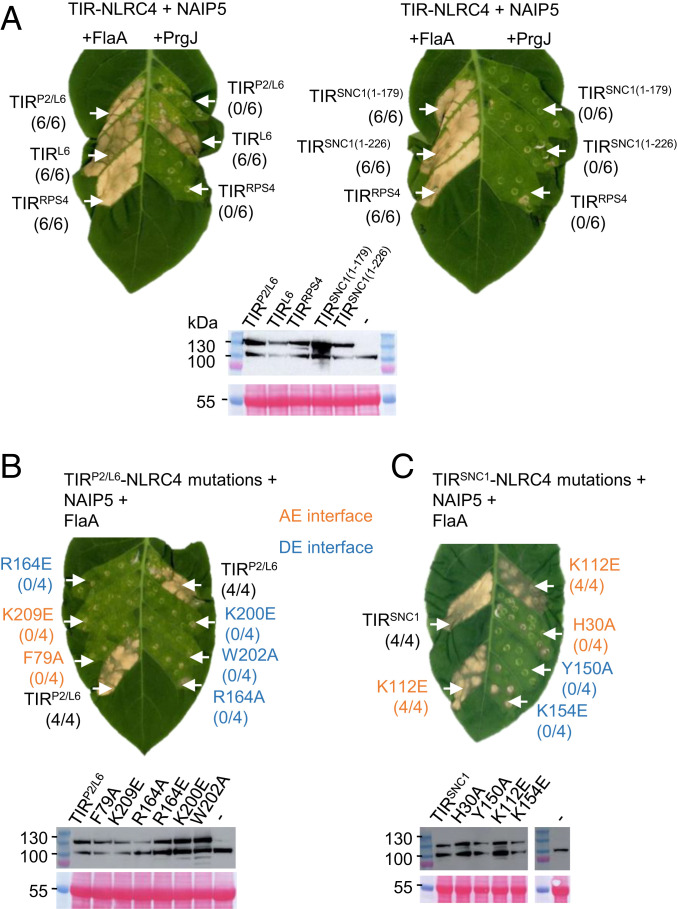
HR phenotype of transiently expressed plant TIR-NLRC4 fusions. (*A*) HR phenotype of NLRC4 fusions of L6, SNC1, or RPS4 TIR domains. *N. tabacum* leaves were coinfiltrated with *A. tumefaciens* strains carrying TIR-NLRC4 fusions, NAIP5, and PAMPs (FlaA or PrgJ). HR was photographed 5 dpi. TIR^P2/L6^ represents a TIR^L6^ construct in which the signal anchor is replaced by the N-terminal sequence from flax NLR P2. Two fragments of TIR^SNC1^ were tested, the minimal TIR domain (residue 1–179) and the autoactive fragment (residue 1–226). The numbers in parentheses are the number of leaves displaying HR equivalent to the image shown out of the total number of leaves infiltrated. (*Lower*) Expression analysis of TIR-NLRC4 fusions. Total proteins from *N. tabacum* leaves with expression of HF tag-fused TIR-NLRC4 proteins were immunoblotted with anti-FLAG antibodies. The dash indicates noninfiltrated *N. tabacum* leaf tissue. Staining of RuBisCO with Ponceau S was used as a loading control. (*B* and *C*) Mutations in self-association interfaces of TIR^P2/L6^ or TIR^SNC1^ (residue 1–226) affect cell death triggered by TIR-NLRC4 oligomerization. *N. tabacum* leaves were coinfiltrated with *A. tumefaciens* strains carrying TIR-NLRC4 or interface mutants, with NAIP5 and FlaA. HR was photographed 5 dpi. Mutations of the AE interface are shown in orange and DE interface in blue. (*Lower*) Expression analysis of TIR-NLRC4 and interface mutants.

Induced proximity of N-terminal signaling domains may be a general mechanism of TIR-NLR signaling activation. To test this, we fused to NLRC4 the TIRs of the plant NLRs SNC1 from *Arabidopsis*; N from tobacco; L6, M, and P from flax; and RRS1, an NLR that acts with RPS4 to confer effector perception in *Arabidopsis* (refs. 23, 24, and 29; alignment of TIRs shown in *SI Appendix*, Fig. S3). Two fragments of TIR^SNC1^ activated HR when fused to NLRC4 coexpressed with NAIP5 and FlaA: TIR^SNC1(1-179)^, the minimal TIR domain containing amino acid residues 1–179, and TIR^SNC1(1-226)^, an autoactive fragment (Fig. 3*A*) (9). TIR^L6^ activated HR when fused to NLRC4 coexpressed with NAIP5 and FlaA (Fig. 3*A*) but was also active in the absence of the appropriate ligand, suggesting autoactivity of this construct. TIR^P2/L6^, an L6 construct with its N-terminal Golgi membrane anchor replaced with the N terminus of the flax NLR P2 (30), showed HR only in the presence of the correct ligand (Fig. 3*A*). AE and DE interface mutations in these TIRs also abolished HR (Fig. 3*B*), except for TIR^SNC1(K112E)^ (Fig. 3*B*). Although this mutation reduces the visible HR induced by TIR^SNC1^, it does not affect self-association in solution and has only a minor effect on ion leakage, suggesting that it only partially impairs function (9). TIR^RRS1^-NLRC4 did not trigger inflammasome-dependent HR (*SI Appendix*, Fig. S2*E*), consistent with RRS1’s “sensor” function in the RRS1/RPS4 pair: RPS4 monitors RRS1 and initiates immune signaling when RRS1’s WRKY domain interacts with effectors (24).

In contrast to TIR^SNC1^ and TIR^L6^, the TIRs from N, M, and P did not trigger HR when fused to NLRC4 and coexpressed with FlaA and NAIP5 (*SI Appendix*, Fig. S4). Tobacco leaf sections infiltrated with TIR^N^-NLRC4, NAIP5, and FlaA displayed yellowing, but TIR^M^-NLRC4 and TIR^P^-NLRC4 fusions were green and healthy (*SI Appendix*, Fig. S4). Therefore, other factors likely influence TIR domain capacity to trigger HR upon induced proximity.

TIR-mediated immunity is dependent on proteins that act as hubs for signaling, including the lipase-like nucleocytoplasmic protein, EDS1, and the RPW8-NLR, NRG1 (2, 31, 32). TIR^RPS4^-NLRC4/NAIP5/FlaA did not trigger HR in an EDS1-silenced tobacco RNA interference (RNAi) line but did trigger HR in nonsilenced tobacco (*SI Appendix*, Fig. S5*A*). We also tested the activation of HR by TIR^RPS4^-NLRC4/NAIP5/FlaA in *N. benthamiana nrg1* mutants. TIR^RPS4^-NLRC4/NAIP5/FlaA triggered HR in WT *N. benthamiana* but not in the *nrg1* mutant (*SI Appendix*, Fig. S5*B*). Furthermore, we demonstrated the requirement of EDS1 for HR triggered by other TIR-NLRC4 constructs by coinfiltrating NAIP5, FlaA, and NLRC4 fusions to either TIR^L6^, TIR^P2/L6^, or TIR^SNC1^ in *N. benthamiana eds1* mutants (*SI Appendix*, Fig. S5*C*). Complementing these mutants transiently with *N. benthamiana* EDS1 rescued HR triggered by these TIR-NLRC4 constructs (*SI Appendix*, Fig. S5*C*) (33). The genetic requirement for the immune signaling hubs EDS1 and NRG1, as well as the requirement for intact TIR interaction interfaces, demonstrates that HR triggered by TIR^RPS4^-NLRC4/NAIP5/FlaA mimics RPS4/RRS1-mediated HR.

The TIRs used in this study (except for that of RRS1) contain a conserved glutamate, which is at position 88 in RPS4 (E88) (*SI Appendix*, Fig. S3). The glutamate at this position is required for TIR NADase activity (26–28). Mutating this residue in full-length RPS4 or TIR^RPS4^ abolishes HR (22, 27, 34). An E88A substitution to TIR^RPS4^-NLRC4 abolished HR upon inflammasome formation (*SI Appendix*, Fig. S2 *B*, *C*, and *E*). We hypothesized that the induced inflammasome would activate plant TIR NADase activity, which is required for HR in our system. To test this hypothesis, we coexpressed the inflammasome components in *N. benthamiana* and purified the TIR-NLRC4 proteins or complexes by immunoprecipitation. Beads with the purified TIR-NLRC4 proteins were then incubated with NAD^+^ (5 µM), and metabolites were extracted and analyzed using liquid chromatography with tandem mass spectrometry (LC-MS/MS). NAD^+^ can be cleaved into nicotinamide (Nam), ADPR, and cADPR (25). A TIR^SARM1^-NLRC4/NAIP5/FlaA inflammasome complex showed strong production of Nam as well as a reduction in NAD^+^ levels in the reaction mix (*SI Appendix*, Fig. S6 *A* and *B*). However, when purified from leaves expressing TIR^SARM1^-NLRC4/NAIP2/FlaA, the monomeric TIR^SARM1^-NLRC4 did not show NADase activity (*SI Appendix*, Fig. S6 *A* and *B*), consistent with a requirement for oligomerization for SARM1-TIR enzymatic activity (26). In contrast, we detected no significant loss of NAD^+^ and no detectable production of Nam with TIR^RPS4^-NLRC4/NAIP5/FlaA or TIR^P2/L6^-NLRC4/NAIP5/FlaA (*SI Appendix*, Fig. S6 *A* and *B*). TIR^L6^ has been reported to have higher enzymatic activity than TIR^RPS4^, but both proteins have much lower activity than detected for SARM1 (28).

Recent reports revealed that plant TIR domains, like certain prokaryotic TIR domains, cleave NAD^+^ into a variant cADPR (v-cADPR), and v-cADPR may serve as a signaling molecule to execute cell death (25, 27). The bacterial *A. baumannii* TIR (TIR^AbTir^) domain, which produces v-cADPR (25), was fused to NLRC4. We first tested its NADase activity after expressing TIR^AbTir^-NLRC4 with NAIPs and FlaA in *N. benthamiana* and immunoprecipitating TIR^AbTir^-NLRC4 and associated proteins. Production of v-cADPR was detected, but no significant depletion of NAD^+^ or production of Nam was detected, from in vitro reaction mixes containing immunoprecipitated protein from extracts from leaves coexpressing either the inflammasome-forming combination of TIR^AbTir^-NLRC4/NAIP5/FlaA, but also in reaction mixes containing TIR^AbTir^-NLRC4/NAIP2/FlaA, a combination that does not form an inflammasome (*SI Appendix*, Fig. S7 *A*–*C*). This suggests that oligomerization is not required for NADase activity in the TIR^AbTir^-NLRC4 fusion. To test whether v-cADPR can signal cell death in planta, we measured v-cADPR production in leaves expressing TIR-NLRC4 fusions. v-cADPR was detected upon transient expression of TIR^AbTir^-NLRC4/NAIP5/FlaA and TIR^AbTir^-NLRC4/NAIP2/FlaA in *N. bethamiana* but not for other TIR-NLRC4 combinations, again demonstrating that TIR^AbTir^-NLRC4 NADase activity is oligomerization-independent (*SI Appendix*, Fig. S7*D*). However, TIR^AbTir^-NLRC4/NAIP5/FlaA did not cause HR in tobacco (*SI Appendix*, Fig. S7*F*). In addition, cADPR accumulated in leaves coexpressing TIR^SARM1^-NLRC4/NAIP5/FlaA, and also in threefold lower levels in leaves coexpressing TIR^SARM1^-NLRC4/NAIP2/FlaA in planta (*SI Appendix*, Fig. S7*E*), but TIR^SARM1^-NLRC4/NAIP5/FlaA also did not induce HR (*SI Appendix*, Fig. S6*C*). Both the results from TIR^AbTir^ and TIR^SARM1^ suggest that neither v-cADPR nor cADPR are sufficient for cell death activation. Our data are consistent with previous results suggesting that plant TIRs have much lower NADase activity than TIR^AbTIR^ and TIR^SARM1^ (27, 28). We infer that plant TIR NADase activity, while essential, is not sufficient for cell death and defense activation in plant cells.

**Fig. 4.**
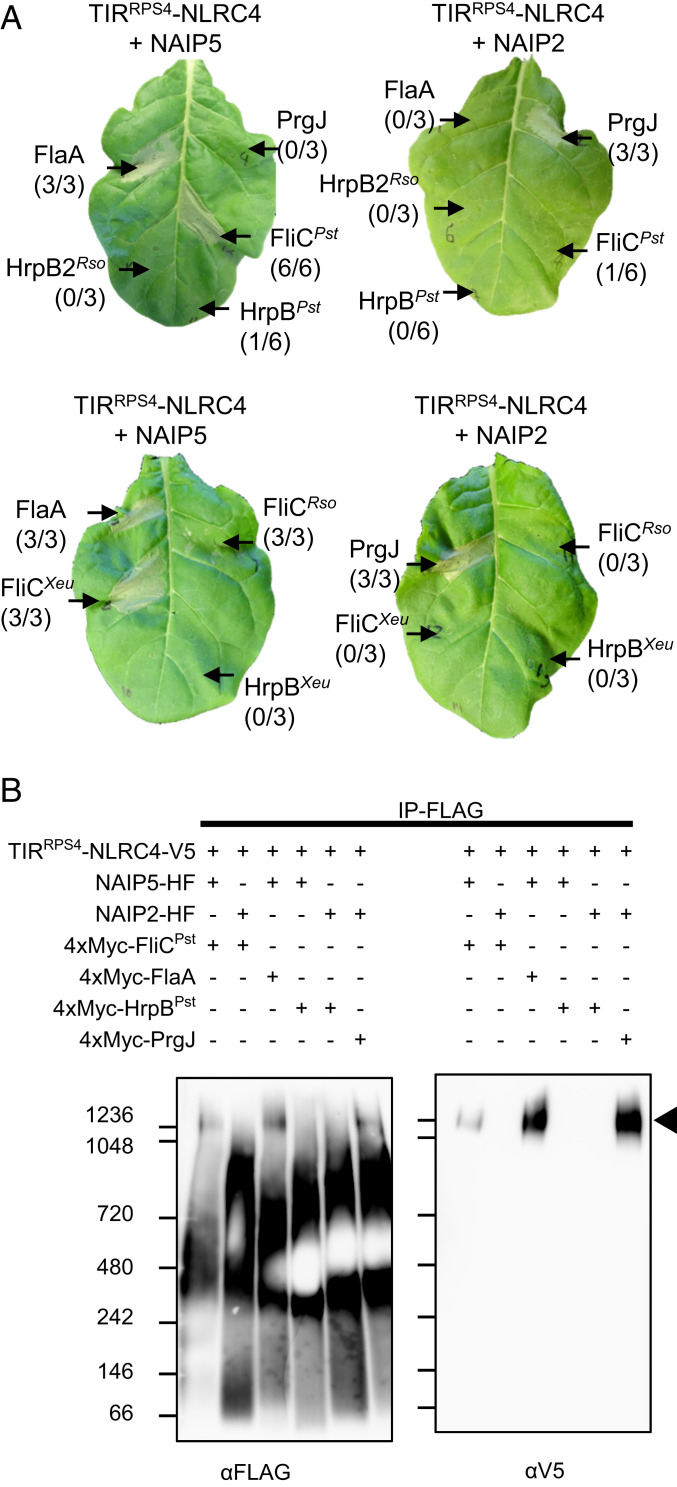
TIR^RPS4^-NLRC4 and NAIP5 confer perception of flagellin from plant-pathogenic bacteria in plants. (*A*) Transient coexpression of TIR^RPS4^-NLRC4 and plant-pathogenic bacterial flagellin and NAIP5, but not NAIP2 and T3SS rod components, triggers HR-like cell death when coexpressed with TIR^RPS4^-NLRC4. *N. tabacum* leaf sections were coinfiltrated with *A. tumefaciens* strains (each at OD_600_ = 0.5) carrying TIR^RPS4^-NLRC4, a NAIP, and a PAMP. HR was visually assessed and photographed after 3 dpi. The numbers in parentheses are the number of leaves displaying HR equivalent to the image shown out of the total number of leaves infiltrated. Superscript abbreviations indicate plant-pathogenic bacterial origin of genes encoding each PAMP: *Pst*, *Pst* DC3000; *Xeu*, *X. euvesicatoria*; *Rso*, *Ralstonia solanacearum*. (*B*) BN-PAGE of immunoprecipitated combinations of TIR^RPS4^-NLRC4, NAIP, and *Pst* DC3000 PAMPs. Arrowheads indicate inflammasome oligomer. See also *SI Appendix*, Fig. S8.

Plants recognize bacterial PAMPs, including flagellin and EF-Tu, via cell-surface receptors. Although bacterial PAMPs enter the plant cytosol during infection, plants lack cytosolic receptors that can detect them (35). We sought to test if TIR^RPS4^-NLRC4/NAIP would function as an intracellular surveillance system in plants for PAMPs secreted by plant-pathogenic bacteria. We generated expression vectors carrying FlaA or PrgJ homologs from the plant-pathogenic bacteria *Pseudomonas syringae* pv. *tomato* (*Pst*) DC3000 (FliC^*Pst*^ and HrpB^*Pst*^), *Ralstonia solanacearum* (FliC^*Rso*^ and HrpB2^*Rso*^), and *Xanthomonas euvesicatoria* (FliC^*Xeu*^ and HrpB^*Xeu*^). FliC^*Pst*^ triggered HR specifically when coexpressed in tobacco with TIR^RPS4^-NLRC4 and NAIP5, while FliC^*Rso*^ and FliC^*Xeu*^ triggered a weaker cell death; the PrgJ homologs HrpB^*Pst*^, HrpB2^*Rso*^, and HrpB^Xeu^ did not trigger HR when coexpressed with TIR^RPS4^-NLRC4 and NAIP2 (Fig. 4*A*). Consistent with these HR phenotypes, FliC^*Pst*^ induced TIR^RPS4^-NLRC4/NAIP5 inflammasome formation but HrpB^*Pst*^ did not induce TIR^RPS4^-NLRC4/NAIP2 inflammasome formation (Fig. 4*B* and *SI Appendix*, Fig. S8). Therefore, the TIR^RPS4^-NLRC4/NAIP inflammasome can assemble and trigger HR in plants in response to flagellin from plant pathogenic bacteria, but not in response to rod components. However, despite reports that bacterial flagellin from *Pst* DC3000 can enter plant cells (35), we were unable to detect enhanced bacterial resistance in transgenic *Arabidopsis* lines carrying TIR^RPS4^-NLRC4 and NAIP5 (*SI Appendix*, Fig. S9). The discrepancy between the observation of HR in transient assays and the lack of an immune response in stably transformed *Arabidopsis* may be due to the different amounts of flagellin present in plant cells in each assay: Overexpression of flagellin in transient assays is likely to result in much more intracellular flagellin accumulation than occurs during *Pst* DC3000 infection.

By fusing plant NLR TIR domains to the N terminus of the mammalian NLR NLRC4, we show here that induced proximity of plant NLR TIRs is sufficient for immune activation, resembling the activation mechanism of mammalian NLR CARDs. These data emphasize the modular nature of NLRs, where the NB domain provides a regulated mechanism for oligomerization and inducing proximity of N-terminal domains, and N-terminal domains can be swapped between NLRs and retain functionality. Similarly, our data demonstrate that the known components of the NAIP/NLRC4 inflammasome can detect ligands and functionally assemble when transplanted across kingdoms, suggesting that there are no additional unidentified mammalian-specific components required for NAIP/NLRC4 activation.

TIR activation required authentic oligomerization at the center of the assembled inflammasome. The linear helix hypothesized to form in an active TIR oligomer (9), or another signaling active conformation, must assemble in the TIR-NLRC4/NAIP5/FlaA inflammasome. Although the structure of the NLRC4 CARDs in an inflammasome has not been resolved, cryoelectron tomography predicts a helix at the center of an inflammasome (36), while purified NLRC4 CARDs in vitro form a tetramer that comprises the base of a helical filament (37, 38). Steric constraints imposed by CARD oligomerization may have prevented the assembly into a signaling-active conformation of some of the plant TIRs we tested.

The assembly of the ZAR1/RKS1/PBL2 resistosome coordinates the ZAR1-CCs into a pore-like structure. This structure resembles a pore-forming toxin, leading to the hypothesis that the ZAR1 resistosome triggers HR cell death through integration and pore formation in the plant plasma membrane (18). Although ZAR1 similarly requires induced proximity of N-terminal signaling domains, the TIR domains of RPS4 and other TNLs are not predicted to form pores.

We were unable to detect plant TIR domain NADase activity in immunopurified plant TIR-NLRC4 complex, perhaps due to its low enzymatic activity and abundance. However, NADase enzymatic activity is a conserved function across prokaryotic and eukaryotic TIR domain proteins (25–28), and the key catalytic glutamate residue is widely present in plant TIR domains (*SI Appendix*, Fig. S3). We infer that NADase activity of plant TIRs and the production of v-cADPR may be necessary but not sufficient for cell death and defense activation. Our data (*SI Appendix*, Fig. S5) and other reports show that plant TIR domain-induced HR requires EDS1, SAG101, and NRG1 (31, 32, 39–42). EDS1-SAG101-NRG1 have coevolved as a module to mediate cell death signaling by TIR-domain immune receptors within plant species (42, 43). Future work will focus on the additional components required for v-cADPR activation of immune signaling.

## Materials and Methods

### Plant Material and Growth Conditions.

*Nicotiana tabacum* cv. “Petite Gerard” or “Samsun” and *N. benthamiana* were grown on soil at 24 °C, 55% relative humidity with a 16/8-h light/dark photoperiod. *Arabidopsis thaliana* accession “Col-0” was grown at 21 °C, 70% humidity with 10/14-h light/dark photoperiod. The *N. tabacum* cv. “Samsun” EDS1 RNAi line was provided by Barbara Baker, Department of Plant and Microbial Biology, University of California, Berkeley, CA, the *N. benthamiana eds1* line was provided by Brian Staskawicz, Department of Plant and Microbial Biology, University of California, Berkeley, CA (33), and we previously generated the *N. benthamina nrg1* line (32).

### Plasmid Construction.

Plasmids were constructed using Golden Gate cloning, as described in refs. 24 and 44. The TIR domain of RPS4 was PCR amplified with primers containing BpiI recognition sites and a specific 4-bp overhang, then cloned into the N-terminal tag module pICSL01002. All other TIR domains were similarly cloned. The TIR^SARM1^ was PCR amplified from pGW1-Myc-Sarm1 (45), a gift from Yi-Ping Hsueh, Institute of Molecular Biology, Academia Sinica, Taipei, Taiwan (Addgene plasmid no. 50707; http://www.addgene.org/50707/; RRID:Addgene_50707). NLRC4, NAIPs, and PAMPs were similarly cloned into the coding sequence module pICSL01005. Modules in pICSL01005 and pICSL01002 were released with BsaI digestion and assembled in pICSL86900 with the CaMV35S promoter, Ocs terminator, and N-terminal and/or C-terminal epitope tags.

### Leaf Infiltration.

Transient transformation by agroinfiltration of *N. tabacum* lamina sections between veins for HR and whole *N. benthamiana* leaves for protein analyses was performed on 4**-** to 5-wk-old plants. *Agrobacterium tumefaciens* strains were mixed in infiltration medium (10 mM MgCl_2_, 10 mM 2-(*N*-morpholino) ethanesulfonic acid [MES], pH 5.6) each at an OD_600_ of 0.5, and hand-infiltrated with a 1-mL needle-less syringe.

### Protein Assays.

Protein was extracted from transiently transformed *N. benthamiana* leaves 72 h postinfiltration (hpi) with *A. tumefaciens* as previously described (23). Briefly, leaves were harvested and ground in liquid nitrogen, and extracted in GTEN buffer (10% glycerol, 100 mM Tris⋅HCl, pH 7.5, 1 mM EDTA, 150 mM NaCl, 5 mM 1,4-dithiothreitol (DTT), 1× cOmplete protease inhibitor mixture [Roche] and 0.2% [vol/vol] Nonidet P-40). Cleared samples were separated into subsamples for input for BN-PAGE, input for SDS-PAGE and samples for immunoprecipitation. Immunoprecipitations were performed for 4 h at 4 °C with gentle agitation, in the presence of 10 μL per 1 mL of protein extract of anti-FLAG M2 affinity Gel (A2220 Sigma-Aldrich), anti-V5 (A7345 Sigma-Aldrich) or anti-Myc (9E10 ThermoFisher). Beads were washed four times in GTEN buffer. FLAG beads were incubated with 150 ng/μL 3XFLAG peptide (Sigma-Aldrich) for 30 min at 4 °C. Other samples were eluted by boiling in SDS-PAGE loading buffer. For BN-PAGE, IP-FLAG eluate and input samples were mixed with 10× BN-PAGE loading buffer, loaded onto Invitrogen NativePAGE 3–12% Bis-Tris Protein Gels, and electrophoresed according to the manufacturer’s instructions. For SDS-PAGE, anti-V5 and anti-Myc beads, as well as IP-FLAG eluate and input samples, were mixed with 3× SDS-PAGE loading buffer and heated for 20 min at 80 °C. After electrophoresis, separated proteins were transferred to Immunobilon-P PVDF (Merck Millipore) membranes for immunoblotting. Membranes were blocked for 2 h in 5% nonfat milk, probed with horseradish peroxidase (HRP)-conjugated antibodies overnight and imaged.

### NADase Assay and LC-MS/MS Metabolite Measurement.

Transiently expressed proteins were extracted from *N. benthamiana* as described above. NADase activity was measured as described in Essuman et al. (26). To be brief, 20 μL anti-FLAG beads with bound protein was mixed with 5 μL of reaction buffer (924 mM NaCl and 6.4× phosphate buffered saline [PBS]), 5 μL of 100 μM NAD^+^ and 20 μL of water. Reactions were either terminated immediately, or after 30 min at 20 °C with periodic vortexing, by the addition of 50 μL of 1 M perchloric acid and incubation on ice for 10 min. Terminated reactions were neutralized by the addition of 16.7 μL of 3 M K_2_CO_3_. Samples were then centrifuged, and 90 μL of supernatant was frozen in liquid nitrogen and stored at −80 °C. NAD^+^ and nicotinamide (Nam) were measured in reaction supernatants by LC-MS/MS. Samples were separated on 100 × 2.1 mm 2.6 μ Kinetex EVO C18 column with guard, whose outflow was attached to a 50 × 2.1 mm 2.6 μ Kinetex F5 column. The high performance liquid chromatography (HPLC) was run with an aqueous solvent of 0.1% formic acid adjusted to pH 6.02 by addition of ammonium hydroxide, with a gradient to 60% methanol. Target compounds were detected by electrospray MS using the 2020 single quadrupole’s dual ion source in ESI mode, with spray chamber conditions of 200 °C heat block, 250 °C desorbation line, 1.5 L·min^−1^ nebulizer gas, and 15 L·min^−1^ drying gas. The instrument collected positive mode scan data from *m*/*z* 100–800 and single ion monitoring data for masses 123.1, 664, 560, and 542 (positive, total even time 0.1 s).

Plant extracts were prepared and analyzed according to Wan et al. (27). Briefly, after transiently transforming *N. benthamiana* leaves 45 hpi with *A. tumefaciences*, leaves were harvested and ground in liquid nitrogen. Samples were extracted with 50% methanol in water and deproteinized with chloroform. The aqueous phase was lyophilized and reconstituted in 5 mM ammonium formate, centrifuged at 13,000 rpm for 10 min, and the supernatant analyzed by LC-MS/MS. Analysis of v-cADPR was on an Acquity ultra performance liquid chromatography (UPLC) attached to a Xevo TQS tandem quadrupole mass spectrometer. Chromatography was exactly the same as for the single quadrupole. *E. coli* lysate of AbTIR was used as a reference for v-cADPR. Spray chamber conditions were 500 °C desolvation temperature, 900 L·hr^−1^ desolvation gas, 150 L·hr^−1^ cone gas, 7 bar nebulizer pressure.

### Bacterial Growth Assay.

*Pst* DC3000 strains were grown on selective King’s B (KB) medium agar plates for 48 h at 28 °C. Bacteria were harvested from the plates and resuspended in infiltration buffer (10 mM MgCl_2_, pH 5.6), and the OD_600_ of the resuspended cell was adjusted to 0.001. Leaves were hand-infiltrated by a needleless syringe. Leaf discs were harvested using a 6-mm cork borer. Two leaf discs per seedling were used as a single treatment, with four replicates sampled after infiltration and eight replicates after 3 days postinfiltration (dpi). Samples were ground and the lysate was diluted in infiltration buffer, and then spotted on selective KB medium plates.

## Supplementary Material

Supplementary File

Supplementary File

## Data Availability

All data are contained in the manuscript, supplemental figures, or Dataset S1.

## References

[1] Duxbury Z.., Pathogen perception by NLRs in plants and animals: Parallel worlds. BioEssays 38, 769–781 (2016). 27339076 10.1002/bies.201600046

[2] Jones J. D. G., Vance R. E., Dangl J. L., Intracellular innate immune surveillance devices in plants and animals. Science 354, aaf6395 (2016). 27934708 10.1126/science.aaf6395

[3] Jones J. D. G., Dangl J. L., The plant immune system. Nature 444, 323–329 (2006). 17108957 10.1038/nature05286

[4] Bernoux M.., Comparative analysis of the flax immune receptors L6 and L7 suggests an equilibrium-based switch activation model. Plant Cell 28, 146–159 (2016). 26744216 10.1105/tpc.15.00303 PMC4746675

[5] Frost D.., Tobacco transgenic for the flax rust resistance gene *L* expresses allele-specific activation of defense responses. Mol. Plant Microbe Interact. 17, 224–232 (2004). 14964536 10.1094/MPMI.2004.17.2.224

[6] Cesari S.., Cytosolic activation of cell death and stem rust resistance by cereal MLA-family CC-NLR proteins. Proc. Natl. Acad. Sci. U.S.A. 113, 10204–10209 (2016). 27555587 10.1073/pnas.1605483113 PMC5018743

[7] Maekawa T.., Coiled-coil domain-dependent homodimerization of intracellular barley immune receptors defines a minimal functional module for triggering cell death. Cell Host Microbe 9, 187–199 (2011). 21402358 10.1016/j.chom.2011.02.008

[8] Williams S. J.., Structural basis for assembly and function of a heterodimeric plant immune receptor. Science 344, 299–303 (2014). 24744375 10.1126/science.1247357

[9] Zhang X.., Multiple functional self-association interfaces in plant TIR domains. Proc. Natl. Acad. Sci. U.S.A. 114, E2046–E2052 (2017). 28159890 10.1073/pnas.1621248114 PMC5347627

[10] Nishimura M. T.., TIR-only protein RBA1 recognizes a pathogen effector to regulate cell death in *Arabidopsis*. Proc. Natl. Acad. Sci. U.S.A. 114, E2053–E2062 (2017). 28137883 10.1073/pnas.1620973114 PMC5347586

[11] Bentham A., Burdett H., Anderson P. A., Williams S. J., Kobe B., Animal NLRs provide structural insights into plant NLR function. Ann. Bot. 119, 827–702 (2017). 27562749 10.1093/aob/mcw171 PMC5378188

[12] Kofoed E. M., Vance R. E., Innate immune recognition of bacterial ligands by NAIPs determines inflammasome specificity. Nature 477, 592–595 (2011). 21874021 10.1038/nature10394 PMC3184209

[13] Zhao Y.., The NLRC4 inflammasome receptors for bacterial flagellin and type III secretion apparatus. Nature 477, 596–600 (2011). 21918512 10.1038/nature10510

[14] Zhang L.., Cryo-EM structure of the activated NAIP2-NLRC4 inflammasome reveals nucleated polymerization. Science 350, 404–409 (2015). 26449474 10.1126/science.aac5789 PMC4640189

[15] Hu Z.., Structural and biochemical basis for induced self-propagation of NLRC4. Science 350, 399–404 (2015). 26449475 10.1126/science.aac5489

[16] Tenthorey J. L.., The structural basis of flagellin detection by NAIP5: A strategy to limit pathogen immune evasion. Science 358, 888–893 (2017). 29146805 10.1126/science.aao1140 PMC5842810

[17] Salvesen G. S., Dixit V. M., Caspase activation: the induced-proximity model. Proc. Natl. Acad. Sci. U.S.A. 96, 10964–10967 (1999). 10500109 10.1073/pnas.96.20.10964 PMC34227

[18] Wang J.., Reconstitution and structure of a plant NLR resistosome conferring immunity. Science 364, eaav5870 (2019). 30948527 10.1126/science.aav5870

[19] Wang J.., Ligand-triggered allosteric ADP release primes a plant NLR complex. Science 364, eaav5868 (2019). 30948526 10.1126/science.aav5868

[20] Wang G.., The decoy substrate of a pathogen effector and a pseudokinase specify pathogen-induced modified-self recognition and immunity in plants. Cell Host Microbe 18, 285–295 (2015). 26355215 10.1016/j.chom.2015.08.004

[21] Hu M., Qi J., Bi G., Zhou J.-M., Bacterial effectors induce oligomerization of immune receptor ZAR1 in vivo. Mol. Plant 13, 793–801 (2020). 32194243 10.1016/j.molp.2020.03.004

[22] Swiderski M. R., Birker D., Jones J. D. G., The TIR domain of TIR-NB-LRR resistance proteins is a signaling domain involved in cell death induction. Mol. Plant Microbe Interact. 22, 157–165 (2009). 19132868 10.1094/MPMI-22-2-0157

[23] Sarris P. F.., A plant immune receptor detects pathogen effectors that target WRKY transcription factors. Cell 161, 1089–1100 (2015). 26000484 10.1016/j.cell.2015.04.024

[24] Ma Y.., Distinct modes of derepression of an *Arabidopsis* immune receptor complex by two different bacterial effectors. Proc. Natl. Acad. Sci. U.S.A. 115, 10218–10227 (2018). 30254172 10.1073/pnas.1811858115 PMC6187137

[25] Essuman K.., TIR domain proteins are an ancient family of NAD^+^-consuming enzymes. Curr. Biol. 28, 421–430.e4 (2018). 29395922 10.1016/j.cub.2017.12.024 PMC5802418

[26] Essuman K.., The SARM1 Toll/Interleukin-1 Receptor domain possesses intrinsic NAD^+^ cleavage activity that promotes pathological axonal degeneration. Neuron 93, 1334–1343.e5 (2017). 28334607 10.1016/j.neuron.2017.02.022 PMC6284238

[27] Wan L.., TIR domains of plant immune receptors are NAD^+^-cleaving enzymes that promote cell death. Science 365, 799–803 (2019). 31439793 10.1126/science.aax1771 PMC7045805

[28] Horsefield S.., NAD^+^ cleavage activity by animal and plant TIR domains in cell death pathways. Science 365, 793–799 (2019). 31439792 10.1126/science.aax1911

[29] Guo H.., Phosphorylation-regulated activation of the *Arabidopsis* RRS1-R/RPS4 immune receptor complex reveals two distinct effector recognition mechanisms. Cell Host Microbe 27, 769–781.e6 (2020). 32234500 10.1016/j.chom.2020.03.008

[30] Takemoto D.., N-terminal motifs in some plant disease resistance proteins function in membrane attachment and contribute to disease resistance. Mol. Plant Microbe Interact. 25, 379–392 (2012). 22046960 10.1094/MPMI-11-10-0272

[31] Qi T.., NRG1 functions downstream of EDS1 to regulate TIR-NLR-mediated plant immunity in *Nicotiana benthamiana*. Proc. Natl. Acad. Sci. U.S.A. 115, E10979–E10987 (2018). 30373842 10.1073/pnas.1814856115 PMC6243234

[32] Castel B.., Diverse NLR immune receptors activate defence via the RPW8-NLR NRG1. New Phytol. 222, 966–980 (2019). 30582759 10.1111/nph.15659

[33] Schultink A., Qi T., Lee A., Steinbrenner A. D., Staskawicz B., Roq1 mediates recognition of the Xanthomonas and Pseudomonas effector proteins XopQ and HopQ1. Plant J. 92, 787–795 (2017). 28891100 10.1111/tpj.13715

[34] Sohn K. H.., The nuclear immune receptor RPS4 is required for RRS1SLH1-dependent constitutive defense activation in *Arabidopsis thaliana*. PLoS Genet. 10, e1004655 (2014). 25340333 10.1371/journal.pgen.1004655 PMC4207616

[35] Wei H.-L., Chakravarthy S., Worley J. N., Collmer A., Consequences of flagellin export through the type III secretion system of *Pseudomonas syringae* reveal a major difference in the innate immune systems of mammals and the model plant *Nicotiana benthamiana*. Cell. Microbiol. 15, 601–618 (2013). 23107228 10.1111/cmi.12059

[36] Diebolder C. A., Halff E. F., Koster A. J., Huizinga E. G., Koning R. I., Cryoelectron tomography of the NAIP5/NLRC4 inflammasome: Implications for NLR activation. Structure 23, 2349–2357 (2015). 26585513 10.1016/j.str.2015.10.001

[37] Matyszewski M.., Cryo-EM structure of the NLRC4^CARD^ filament provides insights into how symmetric and asymmetric supramolecular structures drive inflammasome assembly. J. Biol. Chem. 293, 20240–20248 (2018). 30385506 10.1074/jbc.RA118.006050 PMC6311515

[38] Li Y.., Cryo-EM structures of ASC and NLRC4 CARD filaments reveal a unified mechanism of nucleation and activation of caspase-1. Proc. Natl. Acad. Sci. U.S.A. 115, 10845–10852 (2018). 30279182 10.1073/pnas.1810524115 PMC6205419

[39] Aarts N.., Different requirements for *EDS1* and *NDR1* by disease resistance genes define at least two *R* gene-mediated signaling pathways in *Arabidopsis*. Proc. Natl. Acad. Sci. U.S.A. 95, 10306–10311 (1998). 9707643 10.1073/pnas.95.17.10306 PMC21504

[40] Feys B. J.., *Arabidopsis* SENESCENCE-ASSOCIATED GENE101 stabilizes and signals within an ENHANCED DISEASE SUSCEPTIBILITY1 complex in plant innate immunity. Plant Cell 17, 2601–2613 (2005). 16040633 10.1105/tpc.105.033910 PMC1197438

[41] García A. V.., Balanced nuclear and cytoplasmic activities of EDS1 are required for a complete plant innate immune response. PLoS Pathog. 6, e1000970 (2010). 20617163 10.1371/journal.ppat.1000970 PMC2895645

[42] Lapin D.., A coevolved EDS1-SAG101-NRG1 module mediates cell death signaling by TIR-domain immune receptors. Plant Cell 31, 2430–2455 (2019). 31311833 10.1105/tpc.19.00118 PMC6790079

[43] Gantner J., Ordon J., Kretschmer C., Guerois R., Stuttmann J., An EDS1-SAG101 complex is essential for TNL-mediated immunity in *Nicotiana benthamiana*. Plant Cell 31, 2456–2474 (2019). 31266900 10.1105/tpc.19.00099 PMC6790086

[44] Weber E., Engler C., Gruetzner R., Werner S., Marillonnet S., A modular cloning system for standardized assembly of multigene constructs. PLoS One 6, e16765 (2011). 21364738 10.1371/journal.pone.0016765 PMC3041749

[45] Chen C. Y., Lin C. W., Chang C. Y., Jiang S. T., Hsueh Y. P., Sarm1, a negative regulator of innate immunity, interacts with syndecan-2 and regulates neuronal morphology. J. Cell Biol. 193, 769–784 (2011). 21555464 10.1083/jcb.201008050 PMC3166868

