# Supplementary material for: Induced proximity of a TIR signaling domain on a plant-mammalian NLR chimera activates defense in plants
Source: Proc Natl Acad Sci U S A. 2020 Jul 24;117(31):18832–9. doi: 10.1073/pnas.2001185117 (PMC7414095; doi:10.1073/pnas.2001185117)
Supplement: Supplementary File [file pnas.2001185117.sapp.pdf]

PNAS
www.pnas.org

Supplementary Information for

Induced proximity of a TIR signaling domain on a plant-mammalian NLR chimera activates defense in plants.

Zane Duxbury[†], Shanshan Wang[†], Craig I. MacKenzie, Jeannette L. Tenthorey, Xiaoxiao Zhang, Sung Un Huh, Lanxi Hu, Lionel Hill, Pok Man Ngou, Pingtao Ding, Jian Chen, Yan Ma, Hailong Guo, Baptiste Castel, Panagiotis N. Moschou, Maud Bernoux, Peter N. Dodds, Russell E. Vance, Jonathan D. G. Jones

[†]Z.D. and S.W. contributed equally to this work.

Jonathan D. G. Jones.
Email: jonathan.jones@tsl.ac.uk.

This PDF file includes:

Figures S1 to S9

www.pnas.org/cgi/doi/10.1073/pnas.2001185117

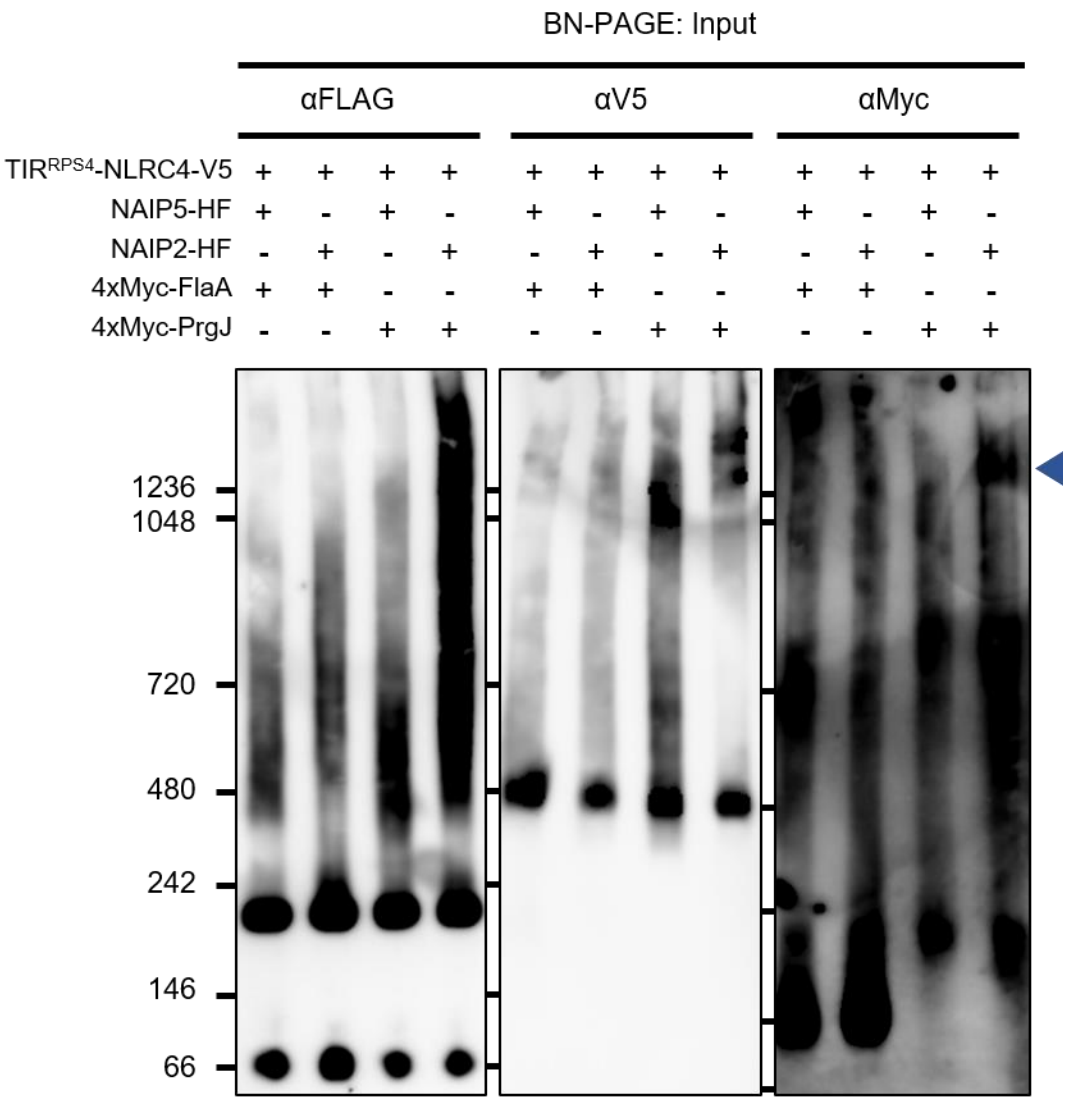

Fig. S1. Input for BN-PAGE in Fig. 1. Blue triangle indicates inflammasome oligomer.

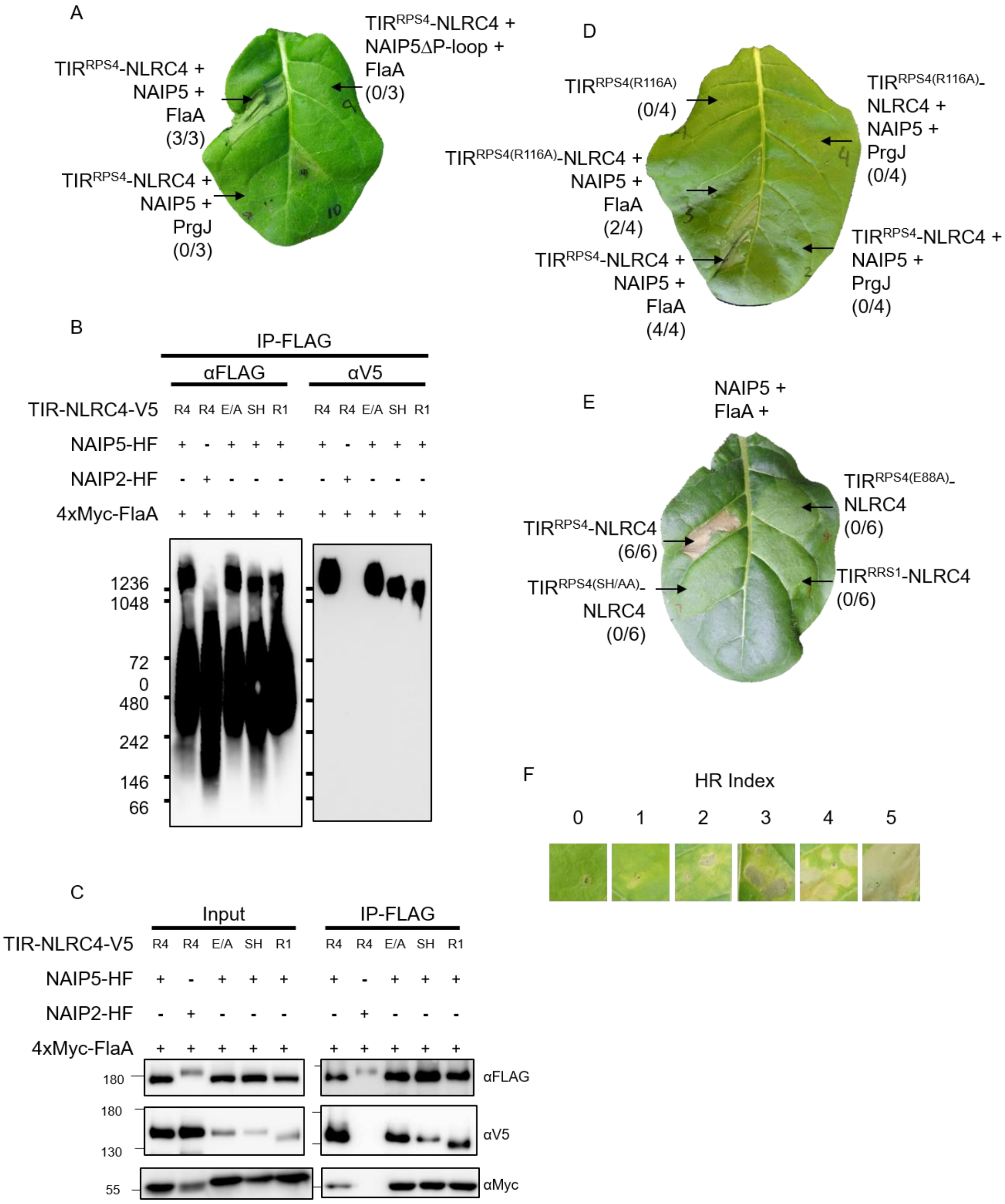

Fig. S2. TIR-domain mutations abolish HR but do not affect inflammasome-like oligomerization. (A) *N. tabacum* leaves were co-infiltrated with *A. tumefaciens* strains (each at OD$_{600}$ = 0.5) carrying TIR^{RPS4}-NLRC4, FlaA and either NAIP5 or a NAIP5 with a deleted P-loop (amino acids 464-487; NAIP5ΔPloop). (B–C) *N. benthamiana* leaves were transiently co-transformed with combinations of TIR-NLRC4, NAIP, PAMP (as indicated by a + or – symbol) and silencing suppressor p19 by *A. tumefaciens* infiltration*.* After 3 days, leaves were harvested, proteins tagged with a FLAG epitope were immunoprecipitated and subjected to BN-PAGE (B) and SDS-PAGE (C), and immunoblotted for V5, FLAG or Myc. TIR-NLRC4-V5 indicates NLRC4-V5 constructs N-terminally fused to one of the following TIR domains: R4, TIR^{RPS4}; E/A, TIR$^{RPS4(E88A)}$; SH, TIR$^{RPS4(SH/AA)}$; R1, TIR^{RRS1} (amino acid residues 1–179). Results shown are representative of three independent replicates. (D) *N. tabacum* leaves were co-infiltrated with NAIP5, FlaA (or PrgJ) and either TIR^{RPS4}-NLRC4 or TIR$^{RPS4(R116A)}$-NLRC4. TIR$^{RPS4(R116A)}$ alone was also infiltrated. (E) *N. tabacum* leaves were co-infiltrated with NAIP5, FlaA and either TIR^{RPS4}-NLRC4, TIR$^{RPS4(SH/AA)}$-NLRC4, TIR$^{RPS4(E88A)}$-NLRC4 or TIR^{RRS1}-NLRC4. HR was visually assessed and

photographed at 3-days post-infiltration (3-dpi). The numbers in parentheses are the number of leaves displaying HR equivalent to the image shown out of the total number of leaves infiltrated. (F) Representative image of HR index used to quantify immune activation.

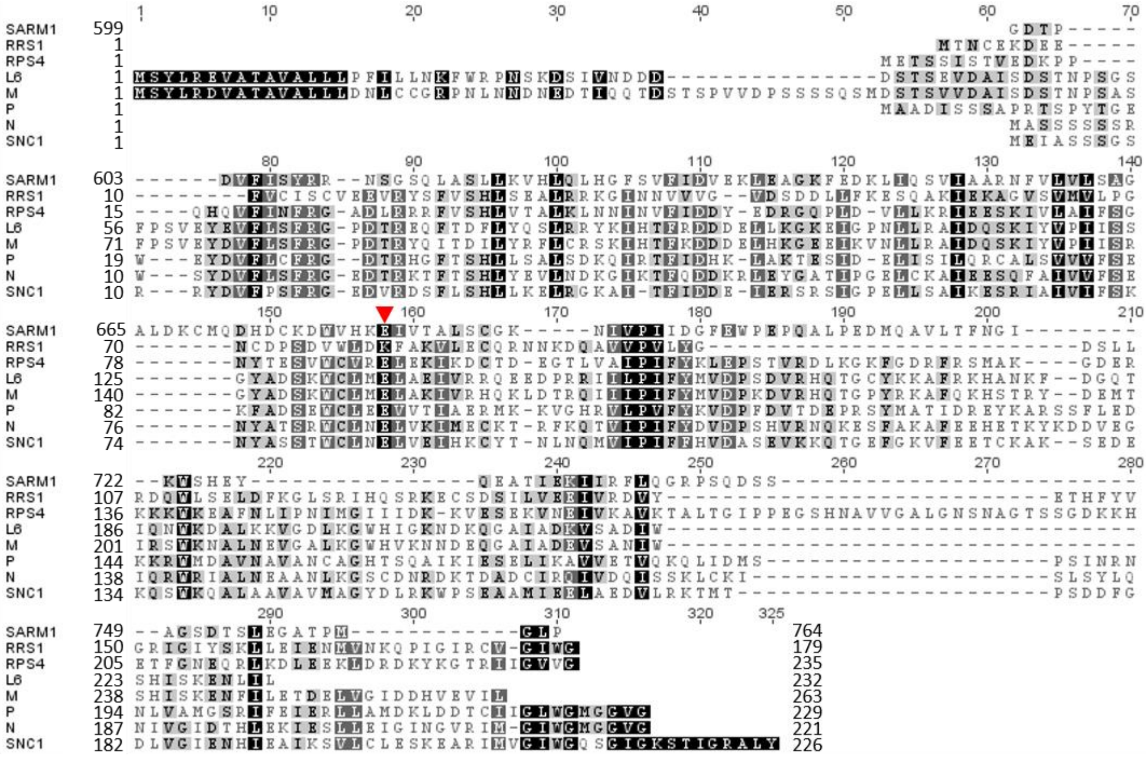

Fig. S3. TIR domain alignment from proteins fused to NLRC4 in this study. Red triangle indicates conserved glutamate required for NADase activity. Box shading is indicative of similarity of amino acids at a position in the alignment. Darker boxes indicate that amino acids in that position are similar and conserved; lighter boxes or no shading indicates less or no similarity or conservation.

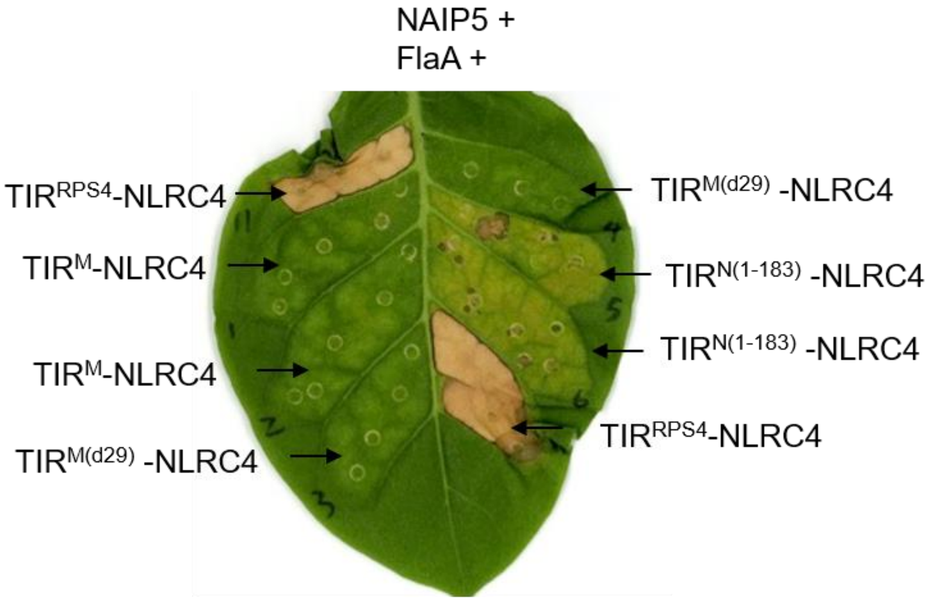
NAIP5 +
FlaA +
TIR^RPS4-NLRC4
TIR^M-NLRC4
TIR^M-NLRC4
TIR^M(d29) -NLRC4
TIR^M(d29) -NLRC4
TIR^N(1-183) -NLRC4
TIR^N(1-183) -NLRC4
TIR^RPS4-NLRC4

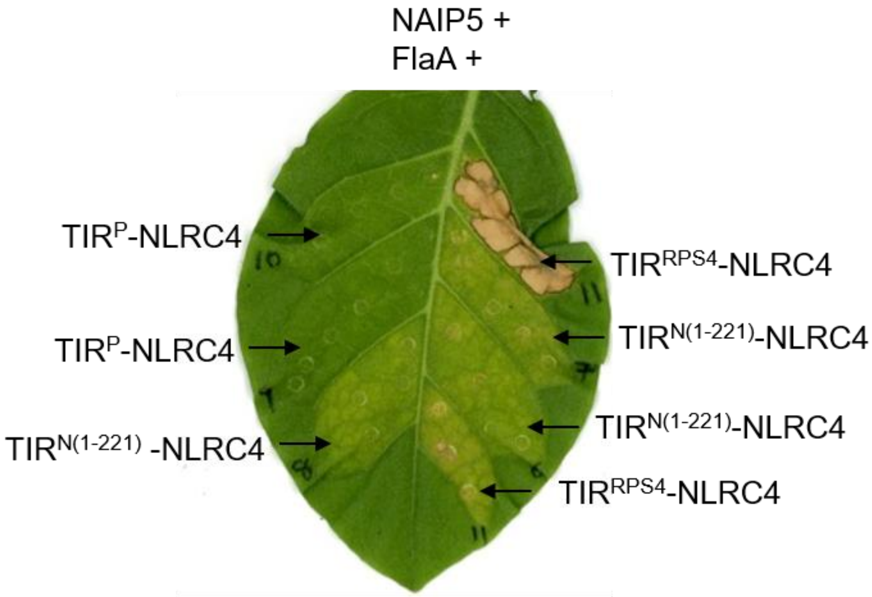
NAIP5 +
FlaA +
TIR^P-NLRC4
TIR^P-NLRC4
TIR^N(1-221) -NLRC4
TIR^RPS4-NLRC4
TIR^N(1-221)-NLRC4
TIR^N(1-221)-NLRC4
TIR^RPS4-NLRC4

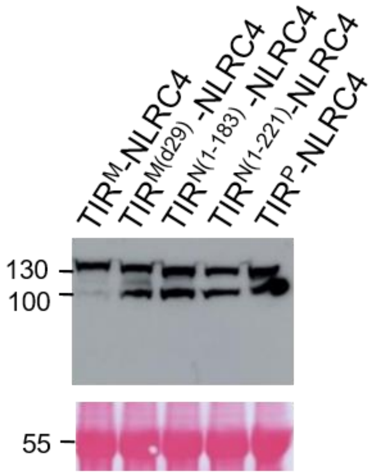
TIR^M-NLRC4
TIR^M(d29) -NLRC4
TIR^N(1-183) -NLRC4
TIR^N(1-221)-NLRC4
TIR^P-NLRC4
130
100
55

Fig. S4. NLRC4 fusions of TIR domains from flax NLRs M and P, and *N. tabacum* NLR N do not trigger cell death when co-expressed with inflammasome components NAIP5 and FlaA. $TIR^{M(d29)}$ represents a TIR^{M} construct with the N-terminal signal anchor deleted. Two fragments of TIR^{N} were tested, the minimal TIR domain (residue 1–183) and a fragment (residue 1–221) equivalent to the autoactive TIR^{SNC1}. Each infiltration was repeated on five independent leaves with equivalent HR phenotypes. Bottom panel: protein expression analysis of TIR-NLRC4 fusions.

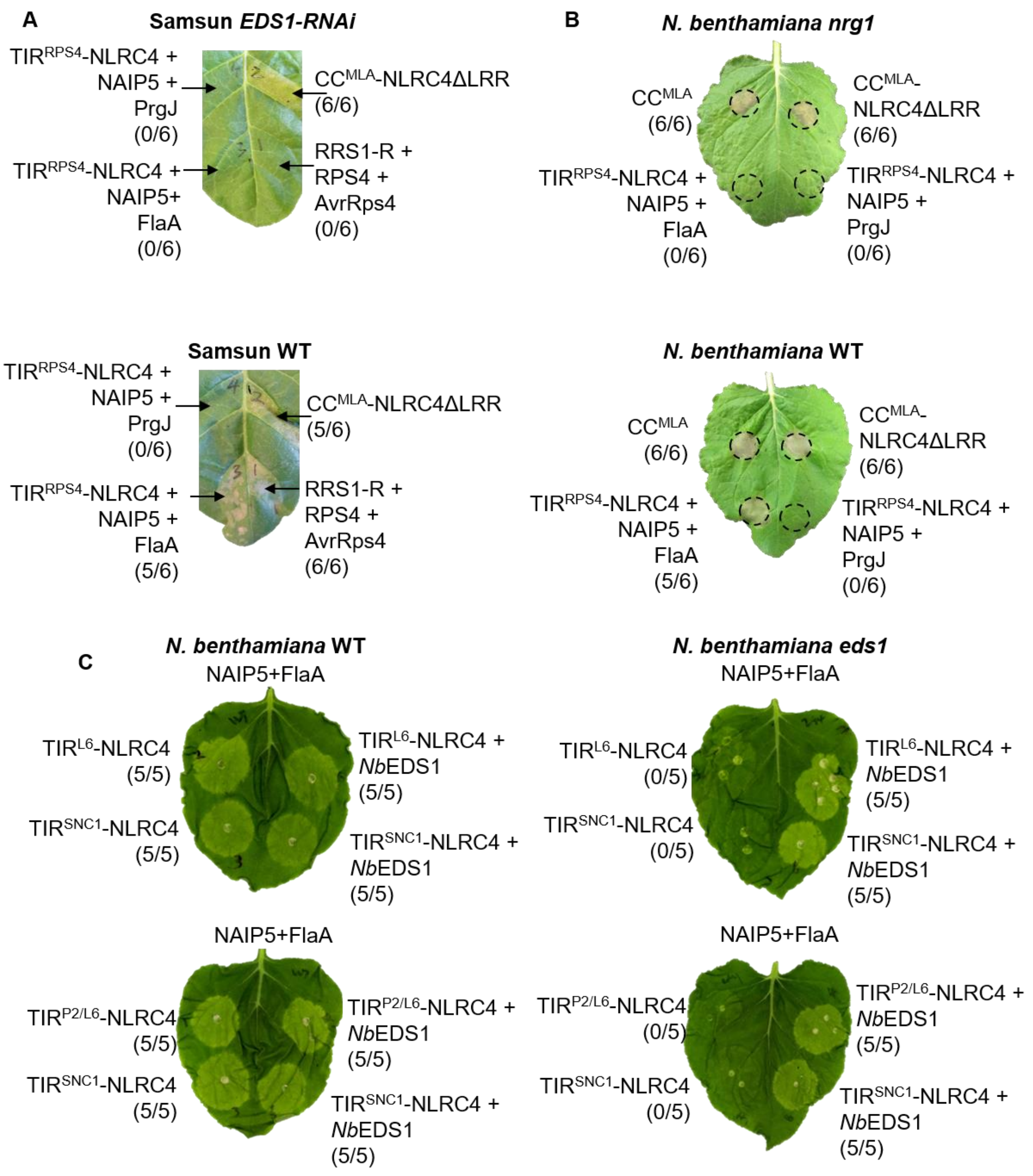

Fig. S5. TIR-NLRC4-mediated HR requires EDS1 and NRG1. (A) Wild-type and an EDS1-suppression line (RNAi) of *N. tabacum* cv. Samsun were co-infiltrated with mixtures of *A. tumefaciens* strains carrying either the inflammasome components TIR^{RPS4}-NLRC4, NAIP5, NAIP2, FlaA or PrgJ, RPS4, RRS1 or AvrRps4 (an effector recognized by RRS1/RPS4) and photographed after 3-dpi. To demonstrate that silenced or mutated plants could support TIR-independent HR, we tested if the N-terminal signaling domain from a coiled-coil (CC)-NLR, another class of NLRs that does not require EDS1 for immune signaling, could trigger HR. CC^{MLA}-NLRC4ΔLRR is an auto-oligomerizing form of NLRC4 fused to the coiled-coil (CC) domain of MLA7 (amino acid residues 1–160). We included CC^{MLA7}-NLRC4ΔLRR as a control because it triggered HR-like cell death in both EDS1-silenced and wild-type tobacco. The numbers in parentheses are the number of leaves displaying HR equivalent to the image shown out of the total number of leaves infiltrated. (B) Wild-type and an *nrg1* mutant of *N. benthamiana*, generated by CRISPR-Cas9, were similarly infiltrated, and additionally infiltrated with a strain of *A.*

tumefaciens carrying CC^{MLA} only. Leaves were photographed after 7 days. Each strain was infiltrated at an OD_{600} of 0.5. (C) Wild-type and an *eds1* mutant of *N. benthamiana* were similarly co-infiltrated with NAIP5, FlaA, and either TIR^{L6}-NLRC4, TIR^{SNC1}-NLRC4 or $TIR^{P2/L6}$-NLRC4. EDS1 cloned from *N. benthamiana (NbEDS1*) was included in some co-infiltrations to rescue HR in *eds1* mutants.

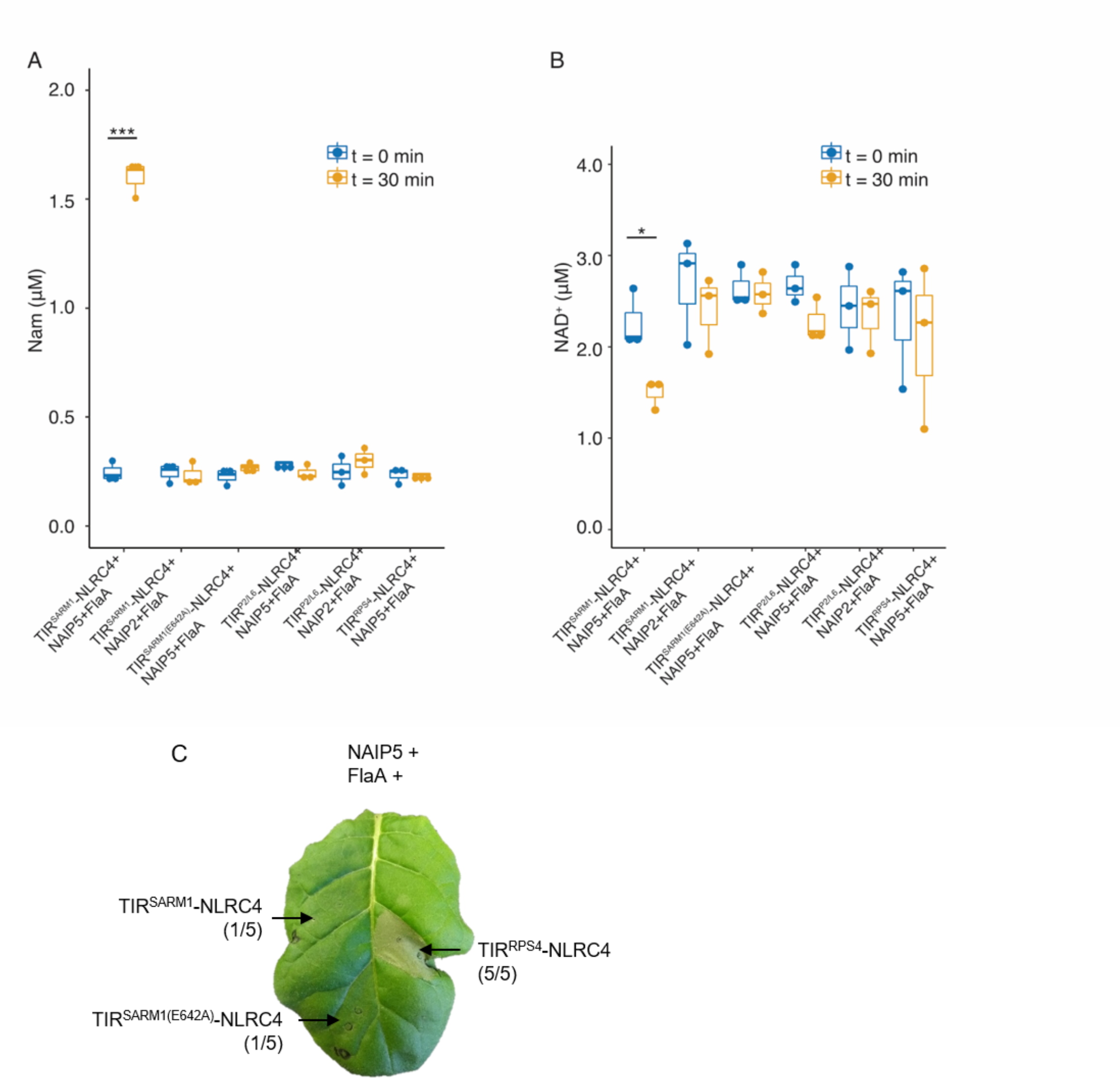

Fig. S6. Plant TIR-NLRC4 inflammasome does not display NADase activity. (A–B) Targeted mass spectrometry measurement of nicotinamide (Nam) and NAD^+ in NADase activity assays. Assays were performed with plant purified TIR-NLRC4 fusion proteins with NAIPs and FlaA. Measurements were taken at 0 and 30 minutes. ****P* < 0.001, * *P* < 0.05, independent two-tailed Student's t test. The experiment was performed three times with similar results. (C) TIR^{SARM1} (residues 599-764) does not trigger HR like cell death when overexpressed in *N. tabacum.* TIR^{SARM1} was expressed either individually or fused to NLRC4 (TIR^{SARM1}). HR was visually assessed at 3-dpi. The numbers in parentheses are the number of leaves displaying HR equivalent to the image shown out of the total number of leaves infiltrated.

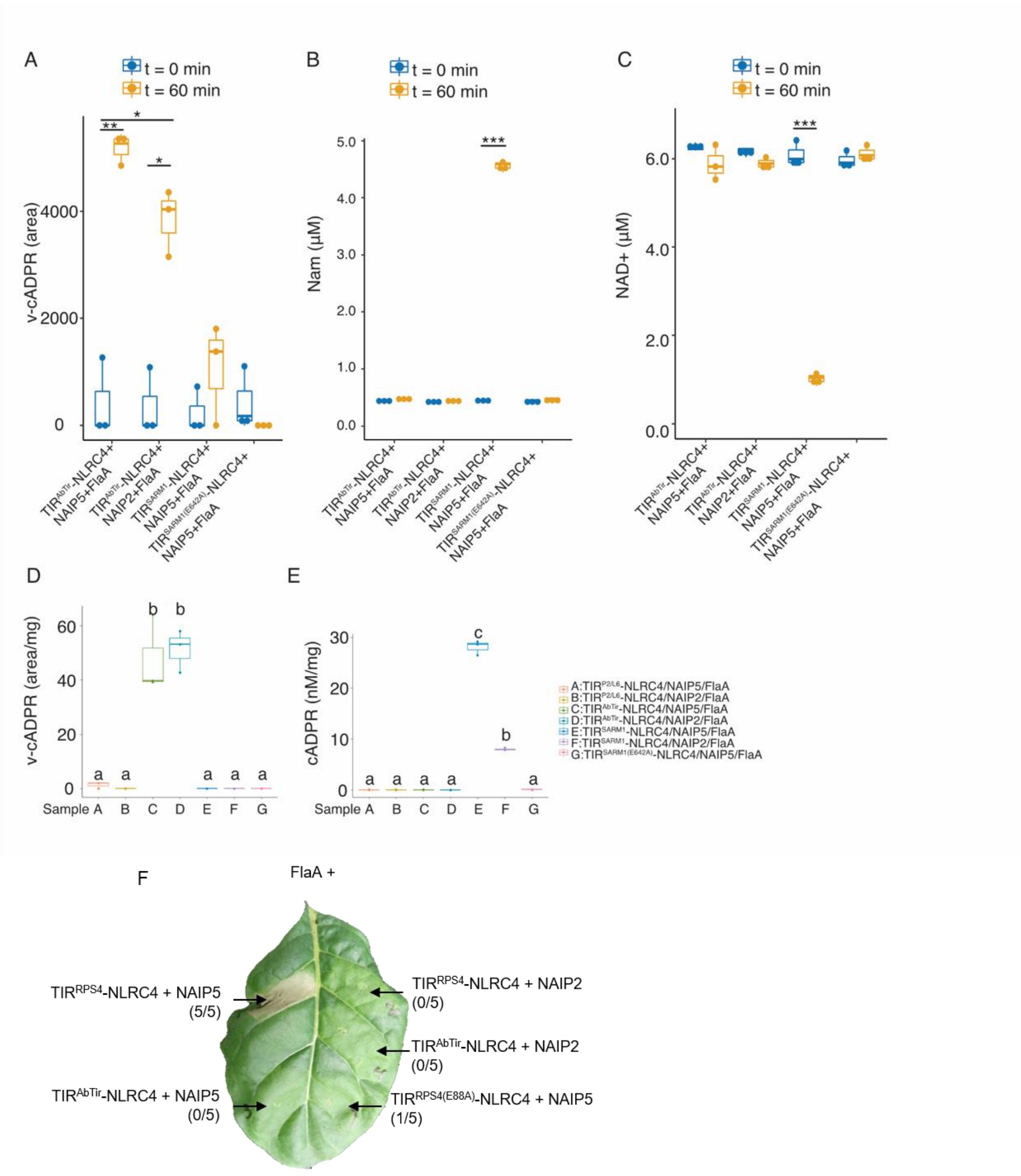

Fig. S7. Variant cADPR is not sufficient to induce HR. (A–C) Targeted mass spectrometry measurement of variant cyclic ADP-ribose (v-cADPR) (A), Nam (B) and NAD^+ (C) produced during *in vitro* NADase activity assays. Assays were performed with purified TIR-NLRC4 fusion proteins with NAIPs and FlaA. Measurements were taken at 0 and 60 minutes. *** $P < 0.001$, *** $P < 0.01$, * $P < 0.05$, independent two-tailed Student's t test. (D-E) Targeted mass spectrometry of v-cADPR and cADPR produced *in planta* and extracted from leaf tissue expressing TIR^{AbTir} fused to NLRC4 with NAIPs and FlaA in *N. benthamiana*. Different letters were significantly different (one-way ANOVA with Duncan test, $P < 0.05$). (A–E) Experiments were performed three times with similar results. (F) TIR^{AbTir} does not trigger cell death when overexpressed in *N. tabacum.* TIR^{AbTir} was fused to NLRC4 and co-expressed with NAIPs and FlaA. HR was visually

assessed at 3-dpi. The numbers in parentheses are the number of leaves displaying HR equivalent to the image shown out of the total number of leaves infiltrated.

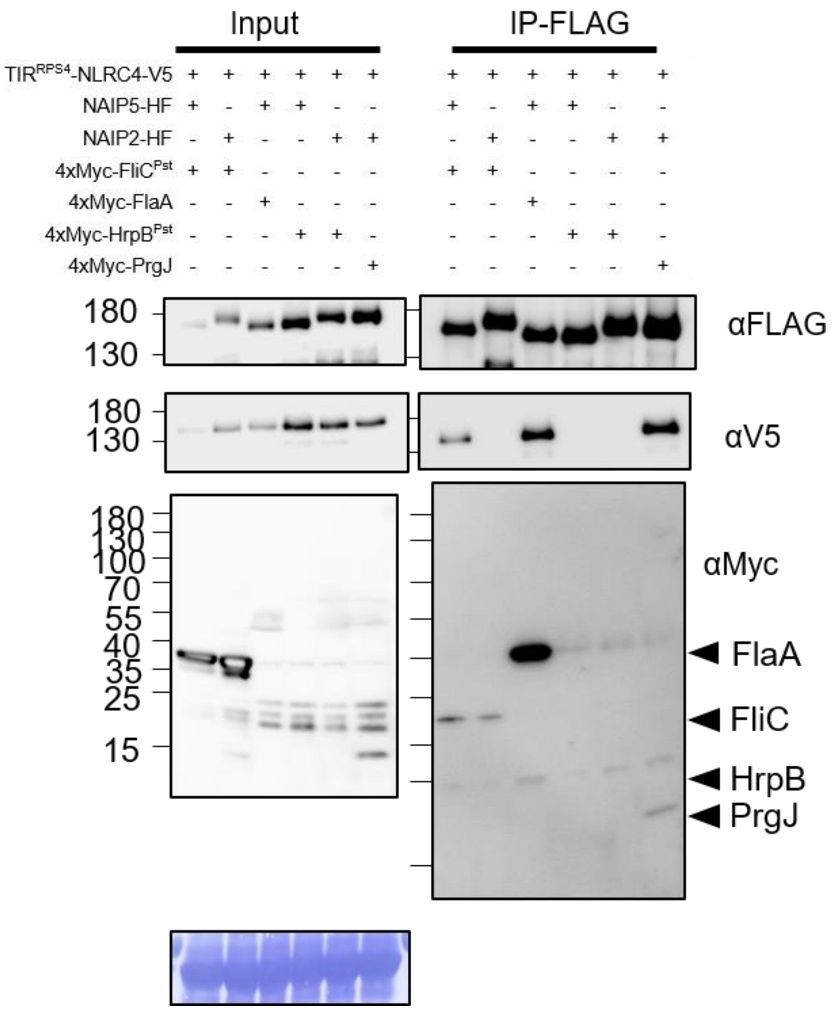

Fig. S8. SDS-PAGE of input and IP-FLAG of combinations of TIR^{RPS4}-NLRC4, NAIP and *Pst* DC3000 PAMPs used in BNG shown in Fig. 4B.

A

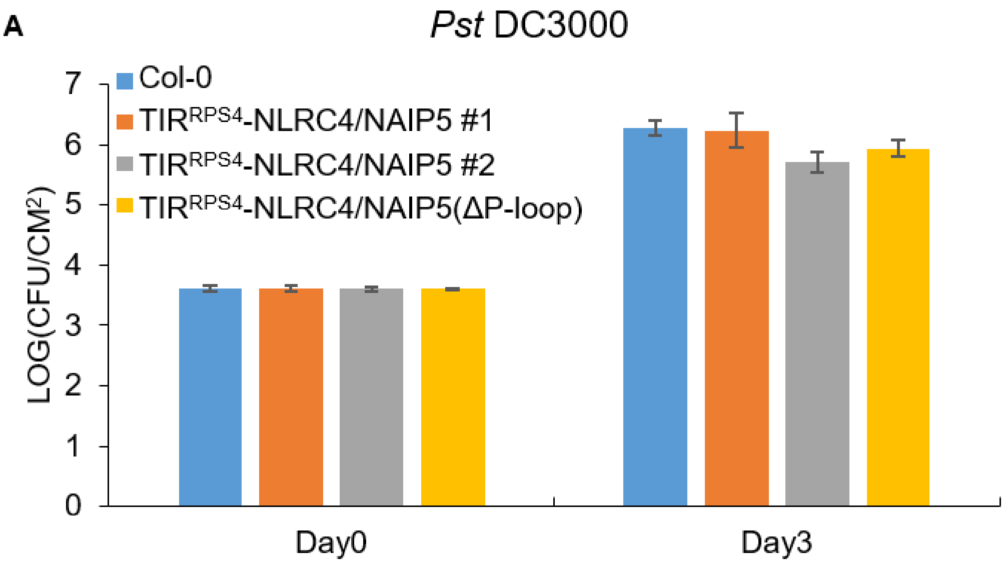

B

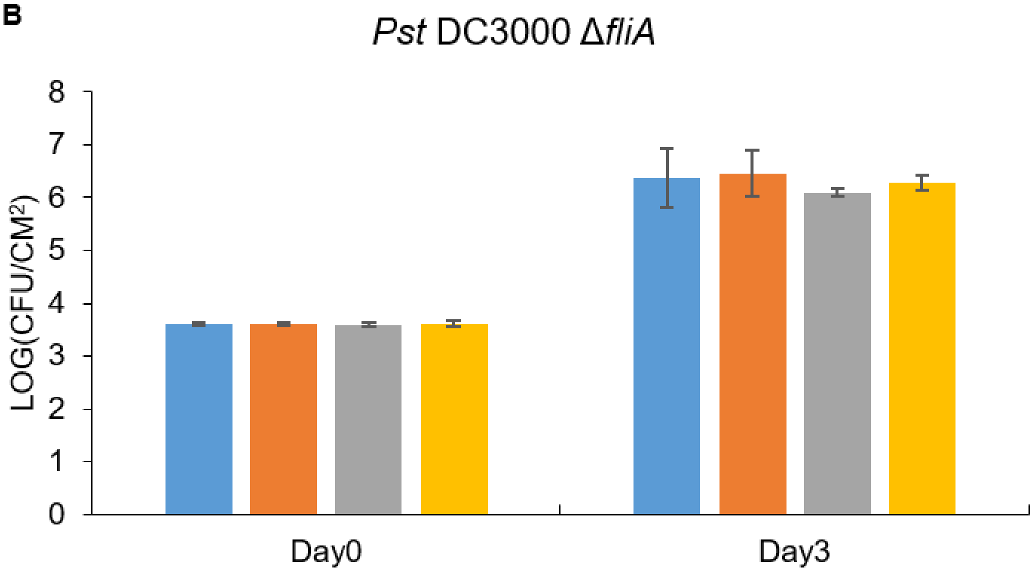

Fig. S9. The TIR^{RPS4}-NLRC4/NAIP5 inflammasome does not confer flagellin-specific bacterial resistance in stable Arabidopsis lines. Bacterial growth assay in Arabidopsis stable lines over-expressing TIR^{RPS4}-NLRC4 and NAIP5 (two lines) or TIR^{RPS4}-NLRC4 and NAIP5ΔP-loop. The growth of bacterial strains *Pseudomonas syringae* pv. *tomato* DC3000 (*Pst* DC3000) (A) or *Pst* DC3000 lacking FliA (*Pst* DC3000ΔFliA) (B) was measured just after infiltration (Day 0) or at 3-days post-infiltration.
